# Supplementary material for: Dynamics of Disagreement: Large-Scale Temporal Network Analysis Reveals Negative Interactions in Online Collaboration
Source: Sci Rep. 2016 Nov 3;6:36333. doi: 10.1038/srep36333 (PMC5093743; doi:10.1038/srep36333)
Supplement: Supplementary Information [file srep36333-s1.pdf]

Supporting Information for:

Dynamics of Disagreement: Large-Scale Temporal Network Analysis Reveals Negative Interactions in Online Collaboration

Milena Tsvetkova, Ruth García-Gavilanes, and Taha Yasseri

Table S1. Results for the counts of the six motifs for 13 different language editions of Wikipedia.

Language	*AB-AB*		*AB-BA*		*AB-BC*		*AB-CB*		*AB-AC*		*AB-CA*	
	**Count data /null**	**Z**	**Count data /null**	**Z**	**Count data /null**	**Z**	**Count data /null**	**Z**	**Count data /null**	**Z**	**Count data /null**	**Z**
English	1018464 /1004006	74.4*	539659 /523510	49.6*	611112 /717717	-204.9*	966610 /988516	-36.3*	1723351 /1779957	-89.0*	1242189 /1185190	76.2*
Spanish	83589 /81208	33.8*	36548 /34373	20.9*	38351 /46190	-39.3*	57297 /58629	-8.1*	131561 /136766	-21.9*	86382 /81164	15.2*
German	28926 /28654	13.2*	24463 /22587	22.9*	10977 /16383	-19.5*	21061 /21390	-4.4*	24235 /25819	-14.2*	21939 /18095	13.5*
Japanese	59833 /59539	10.7*	21562 /23032	-13.2*	11333 /19166	-49.2*	25581 /26784	-12.5*	32332 /35026	-16.8*	24039 /21739	11.0*
French	35759 /35400	11.9*	17180 /16297	12.7*	9076 /12904	-22.6*	18949 /19375	-5.3*	26677 /28883	-15.6*	18142 /15755	11.8*
Portuguese	56504 /54762	26.1*	28549 /27038	14.9*	24935 /29715	-29.8*	34839 /35727	-5.0*	82530 /86292	-21.2*	53664 /51893	7.2*
Chinese	46274 /45704	14.7*	22779 /23113	-5.7*	15918 /21862	-24.3*	23773 /25127	-8.9*	44061 /47358	-17.3*	30481 /29103	7.5*
Hebrew	22460 /21770	23.3*	11645 /10530	24.8*	13423 /16279	-25.7*	19189 /19691	-4.4*	33904 /35734	-15.0*	23947 /22528	11.8*
Arabic	7857 /7750	8.7*	3676 /3511	6.4*	2656 /3483	-24.4*	4276 /4410	-2.2	11901 /12538	-15.4*	5288 /4893	5.1*
Hungarian	6144 /6065	6.6*	2535 /2304	6.1*	1853 /2523	-23.2*	3508 /3640	-2.4	6734 /7116	-9.6*	3636 /3235	9.7*
Persian	11642 /11389	13.0*	7859 /7236	13.0*	7358 /9079	-22.8*	10045 /10152	-1.4	17512 /18319	-8.8*	12522 /11722	6.2*
Czech	5097 /5035	6.5*	2524 /2308	10.2*	1806 /2400	-17.9*	3297 /3236	1.3	4975 /5144	-4.8*	3129 /2842	6.6*
Romanian	4197 /4106	10.2*	1412 /1418	-0.2	999 /1369	-10.8*	1861 /1963	-2.4	5759 /5889	-2.9*	2758 /2527	4.0*

* 2-sided p-value < 0.01

Table S2. Results for the response rate of the six motifs for 13 different language editions of Wikipedia.

Language	*AB-AB*					*AB-BA*					*AB-BC*				
	**KS**	**Mean data /null**	**Mean Z**	**Skew data /null**	**Skew Z**	**KS**	**Mean data /null**	**Mean Z**	**Skew data /null**	**Skew Z**	**KS**	**Mean data /null**	**Mean Z**	**Skew data /null**	**Skew Z**
English	0.20*	100 /221	-459.5*	3.26 /1.79	963.8*	0.08*	241 /313	-99.0*	1.69 /1.27	98.1*	-0.13*	514 /404	142.6*	0.52 /0.88	-97.4*
Spanish	0.25*	81 /226	-184.3*	3.72 /1.75	343.9*	0.09*	242 /327	-49.4*	1.66 /1.2	55.3*	-0.16*	511 /381	64.3*	0.53 /0.97	-47.2*
German	0.10*	128 /190	-47.0*	2.81 /2.06	59.4*	0.12*	195 /285	-37.9*	2.04 /1.44	41.4*	-0.19*	544 /381	50.8*	0.45 /1.00	-36.6*
Japanese	0.13*	56 /122	-112.0*	4.62 /2.81	177.6*	-0.16*	274 /239	13.4*	1.44 /1.67	-11.8*	-0.30*	513 /295	62.3*	0.56 /1.34	-41.7*
French	0.12*	85 /159	-69.2*	3.65 /2.37	98.6*	0.07*	211 /275	-19.5*	1.95 /1.5	24.6*	-0.19*	560 /390	40.3*	0.38 /0.95	-28.6*
Portuguese	0.26*	89 /238	-112.3*	3.53 /1.68	195.1*	0.10*	210 /306	-32.1*	1.89 /1.3	36.3*	-0.19*	510 /363	60.7*	0.56 /1.05	-43.5*
Chinese	0.19*	91 /184	-83.2*	3.47 /2.08	133.1*	-0.15*	300 /273	8.7*	1.32 /1.46	-8.0*	-0.26*	557 /336	45.5*	0.35 /1.15	-34.7*
Hebrew	0.24*	76 /230	-78.3*	3.88 /1.70	145.5*	0.25*	151 /336	-29.7*	2.47 /1.15	47.7*	-0.16*	516 /403	28.4*	0.52 /0.88	-19.8*
Arabic	0.18*	125 /233	-36.7*	2.80 /1.69	47.4*	-0.07*	319 /354	-5.2*	1.22 /1.07	4.5*	-0.22*	623 /431	22.5*	0.17 /0.77	-15.5*
Hungarian	0.15*	75 /173	-39.3*	3.90 /2.20	61.6*	0.08*	229 /312	-11.8*	1.80 /1.29	13.3*	-0.22*	594 /417	16.1*	0.29 /0.84	-11.9*
Persian	0.24*	126 /267	-64.3*	2.82 /1.49	86.1*	0.19*	222 /361	-30.9*	1.81 /1.04	40.3*	-0.15*	532 /399	22.3*	0.47 /0.90	-16.0*
Czech	0.16*	76 /173	-33.0*	3.90 /2.20	55.7*	0.07*	234 /280	-4.4*	1.79 /1.46	5.6*	-0.22*	566 /401	15.9*	0.38 /0.89	-10.3*
Romanian	0.19*	59 /181	-37.6*	4.61 /2.16	73.4*	-0.09*	211 /264	-4.7*	1.90 /1.57	4.2*	-0.24*	487 /324	14.0*	0.63 /1.24	-10.0*

* 2-sided p-value < 0.01

… continued

Language	*AB-CB*					*AB-AC*					*AB-CA*				
	**KS**	**Mean data /null**	**Mean Z**	**Skew data /null**	**Skew Z**	**KS**	**Mean data /null**	**Mean Z**	**Skew data /null**	**Skew Z**	**KS**	**Mean data /null**	**Mean Z**	**Skew data /null**	**Skew Z**
English	0.09*	374 /439	-126.9*	1.03 /0.78	230.8*	-0.12*	380 /323	146.0*	1.00 /1.23	-132.9*	-0.05*	410 /428	-15.1*	0.94 /0.83	26.4*
Spanish	0.07*	406 /464	-29.7*	0.89 /0.68	27.6*	-0.15*	379 /303	40.3*	1.00 /1.32	-34.1*	-0.10*	459 /443	4.5*	0.75 /0.75	-0.4
German	0.02*	364 /381	-6.9*	1.10 /1.01	8.0*	-0.11*	492 /402	36.7*	0.60 /0.91	-28.4*	0.04*	357 /393	-8.8*	1.13 /0.95	10.9*
Japanese	-0.02*	338 /345	-2.2	1.16 /1.12	2.1	-0.21*	413 /293	37.0*	0.86 /1.36	-28.3*	-0.17*	413 /350	10.0*	0.87 /1.08	-7.6*
French	0.05*	355 /388	-8.4*	1.11 /0.97	8.2*	-0.18*	487 /364	44.0*	0.62 /1.05	-34.3*	-0.10*	418 /406	1.8	0.88 /0.89	-0.2
Portuguese	0.04*	442 /478	-10.6*	0.77 /0.64	10.2*	-0.16*	377 /293	58.8*	1.05 /1.39	-39.9*	-0.09*	483 /454	8.2*	0.67 /0.72	-3.5*
Chinese	-0.05*	437 /418	2.5	0.75 /0.8	-1.3	-0.14*	396 /300	39.8*	0.92 /1.32	-31.1	-0.16*	446 /405	8.2*	0.78 /0.87	-4.7*
Hebrew	0.10*	393 /461	-14.6*	0.95 /0.69	14.6*	-0.15*	441 /361	29.8*	0.77 /1.04	-22.9*	-0.04*	443 /453	-2.6*	0.77 /0.70	4.2*
Arabic	-0.05*	509 /504	0.8	0.58 /0.57	0.5	-0.09*	400 /331	17.3*	0.89 /1.16	-15.0*	-0.11*	515 /493	2.5	0.58 /0.56	0.7
Hungarian	0.05*	404 /430	-4.1*	0.89 /0.79	3.1*	-0.13*	431 /353	23.9*	0.79 /1.08	-20.1*	-0.05*	438 /450	-1.0	0.81 /0.72	1.9
Persian	0.05*	402 /446	-9.7*	0.93 /0.74	9.3*	-0.13*	404 /338	18.2*	0.91 /1.15	-13.6*	-0.04*	419 /441	-2.9*	0.86 /0.75	4.3*
Czech	0.07*	376 /420	-3.4*	0.99 /0.82	4.0*	-0.19*	509 /393	15.7*	0.55 /0.93	-10.8*	-0.05*	444 /451	-0.4	0.79 /0.72	1.3
Romanian	0.09*	371 /428	-4.2*	1.07 /0.79	3.9*	-0.17*	360 /282	17.6*	1.13 /1.46	-11.8*	-0.09*	460 /451	0.6	0.78 /0.76	0.2

* 2-sided p-value < 0.01

Table S3. Results for the status difference in the six motifs for 13 different language editions of Wikipedia.

Language	*AB-AB*		*AB-BA*		*AB-BC*			*AB-CB*			*AB-AC*			*AB-CA*		
	**Other**	***A-B***	**Other**	***A-B***	**Other**	***A-B***	***B-C***	**Other**	***A-B***	***C-B***	**Other**	***A-B***	***A-C***	**Other**	***A-B***	**C-A**
English	1.069*	0.121*	1.396*	-1.108*	1.428*	-1.723*	-0.173*	1.293*	-0.485*	-0.282*	0.535*	1.078*	0.648*	1.277*	0.075	-1.460*
	(0.023)	(0.024)	(0.026)	(0.023)	(0.025)	(0.028)	(0.017)	(0.025)	(0.024)	(0.021)	(0.017)	(0.030)	(0.010)	(0.019)	(0.033)	(0.020)
Spanish	1.223*	0.338*	1.629*	-1.119*	1.674*	-2.134*	-0.086	1.554*	-0.647*	-0.495*	0.675*	1.240*	0.839*	1.493*	0.220	-1.818*
	(0.082)	(0.079)	(0.077)	(0.103)	(0.075)	(0.126)	(0.058)	(0.081)	(0.133)	(0.054)	(0.077)	(0.101)	(0.038)	(0.051)	(0.152)	(0.084)
German	0.789*	0.025	1.022*	-0.719*	0.881*	-0.933*	-0.363*	0.795*	0.019	0.007	0.720*	0.331*	0.301*	0.946*	-0.602*	-0.755*
	(0.039)	(0.046)	(0.041)	(0.044)	(0.041)	(0.060)	(0.049)	(0.042)	(0.047)	(0.031)	(0.036)	(0.066)	(0.040)	(0.039)	(0.051)	(0.044)
Japanese	0.636*	0.088	0.855*	-0.769*	0.773*	-1.034*	-0.135	0.661*	0.158*	0.098	0.587*	0.367*	0.380*	0.813*	-0.520*	-0.671*
	(0.059)	(0.061)	(0.070)	(0.071)	(0.067)	(0.107)	(0.078)	(0.069)	(0.061)	(0.061)	(0.055)	(0.091)	(0.056)	(0.061)	(0.098)	(0.090)
French	1.026*	0.192*	1.348*	-1.045*	1.219*	-1.282*	-0.339*	1.097*	0.091	0.009	0.953*	0.547*	0.445*	1.271*	-0.555*	-1.123*
	(0.057)	(0.042)	(0.058)	(0.057)	(0.059)	(0.076)	(0.060)	(0.063)	(0.039)	(0.036)	(0.050)	(0.091)	(0.043)	(0.056)	(0.074)	(0.064)
Portuguese	1.338*	0.013	1.699*	-1.301*	1.695*	-2.201*	-0.057	1.552*	-0.690*	-0.507*	0.580*	1.344*	0.960*	1.504*	0.148	-1.870*
	(0.125)	(0.090)	(0.098)	(0.164)	(0.092)	(0.162)	(0.075)	(0.107)	(0.174)	(0.089)	(0.127)	(0.104)	(0.062)	(0.087)	(0.109)	(0.127)
Chinese	0.992*	-0.146	1.200*	-0.966*	1.134*	-1.508*	-0.016	1.049*	-0.441*	-0.438*	0.503*	0.966*	0.661*	1.053*	-0.069	-1.241*
	(0.226)	(0.122)	(0.186)	(0.100)	(0.168)	(0.192)	(0.120)	(0.192)	(0.158)	(0.133)	(0.101)	(0.258)	(0.112)	(0.119)	(0.256)	(0.173)
Hebrew	1.081*	0.177	1.416*	-0.987*	1.438*	-1.608*	-0.240	1.255*	-0.281	-0.259*	0.700*	0.909*	0.639*	1.373*	-0.145	-1.380*
	(0.132)	(0.078)	(0.119)	(0.111)	(0.113)	(0.143)	(0.094)	(0.125)	(0.140)	(0.084)	(0.120)	(0.100)	(0.057)	(0.110)	(0.111)	(0.117)
Arabic	1.450*	-0.167	1.648*	-1.401*	1.562*	-1.718*	-0.384*	1.446*	-0.257	-0.337*	0.925*	0.899*	0.731*	1.583*	-0.362*	-1.665*
	(0.163)	(0.077)	(0.139)	(0.148)	(0.144)	(0.143)	(0.083)	(0.164)	(0.105)	(0.086)	(0.112)	(0.149)	(0.083)	(0.134)	(0.107)	(0.141)
Hungarian	1.241*	-0.018	1.440*	-1.007*	1.379*	-1.499*	-0.465*	1.260*	-0.068	-0.109	0.969*	0.775*	0.516*	1.399*	-0.403	-1.304*
	(0.157)	(0.084)	(0.144)	(0.131)	(0.143)	(0.136)	(0.087)	(0.156)	(0.134)	(0.111)	(0.110)	(0.212)	(0.085)	(0.131)	(0.165)	(0.113)
Persian	1.096*	0.040	1.525*	-1.190*	1.478*	-1.617*	-0.528*	1.304*	-0.507*	-0.537*	0.644*	0.782*	0.544*	1.513*	-0.580*	-1.573*
	(0.163)	(0.092)	(0.135)	(0.107)	(0.140)	(0.126)	(0.077)	(0.164)	(0.118)	(0.075)	(0.159)	(0.105)	(0.069)	(0.133)	(0.109)	(0.119)
Czech	1.004*	0.292	1.319*	-0.881*	1.239*	-1.289*	-0.246	1.097*	0.083	-0.022	0.849*	0.797*	0.546*	1.279*	-0.481	-1.115*
	(0.163)	(0.113)	(0.164)	(0.147)	(0.169)	(0.202)	(0.121)	(0.187)	(0.100)	(0.088)	(0.122)	(0.208)	(0.123)	(0.151)	(0.197)	(0.151)
Romanian	1.614*	-0.126	1.800*	-1.091*	1.761*	-2.429*	-0.057	1.631*	-0.240	-0.323	0.958*	1.195*	0.957*	1.713*	0.011	-1.965*
	(0.335)	(0.195)	(0.242)	(0.195)	(0.237)	(0.380)	(0.185)	(0.275)	(0.211)	(0.283)	(0.217)	(0.226)	(0.203)	(0.222)	(0.271)	(0.415)

* 2-sided p-value < 0.01
The table reports coefficients from ordinary least-square regression predicting the difference in the log with base ten of the number of edits completed by the time of the revert by the reverter and the reverted individual. The standards errors (in brackets) account for two-way clustering within reverter and reverted.

Table S4. Results for the proportion of the six motifs that occurred in the same article for 13 different language editions of Wikipedia.

Language	*AB-AB*		*AB-BA*		*AB-BC*		*AB-CB*		*AB-AC*		*AB-CA*	
	**Prop. data /rand**	**Z**	**Prop. data /rand**	**Z**	**Prop. data /rand**	**Z**	**Prop. data /rand**	**Z**	**Prop. data /rand**	**Z**	**Prop. data /rand**	**Z**
English	0.222 /0.180	137.3*	0.685 /0.180	1043.7*	0.098 /0.180	-188.7*	0.171 /0.180	-26.6*	0.055 /0.180	-690.0*	0.154 /0.180	-95.5*
Spanish	0.204 /0.150	53.7*	0.677 /0.150	317.5*	0.075 /0.150	-46.1*	0.155 /0.149	4.8*	0.029 /0.150	-199.7*	0.107 /0.150	-45.2*
German	0.411 /0.451	-19.1*	0.884 /0.451	179.3*	0.289 /0.451	-38.9*	0.359 /0.451	-30.2*	0.185 /0.450	-101.1*	0.476 /0.451	8.3*
Japanese	0.094 /0.181	-86.5*	0.572 /0.182	173.6*	0.098 /0.182	-23.1*	0.124 /0.181	-26.1*	0.054 /0.181	-73.5*	0.212 /0.182	13.1*
French	0.231 /0.274	-27.5*	0.762 /0.273	160.0*	0.179 /0.273	-22.8*	0.240 /0.274	-12.6*	0.082 /0.274	-76.8*	0.291 /0.274	5.3*
Portuguese	0.227 /0.159	54.5*	0.652 /0.158	251.8*	0.069 /0.159	-48.6*	0.151 /0.159	-4.2*	0.029 /0.159	-154.3*	0.103 /0.159	-44.2*
Chinese	0.136 /0.167	-28.7*	0.528 /0.166	173.9*	0.069 /0.167	-37.5*	0.112 /0.166	-29.4*	0.038 /0.166	-95.4*	0.128 /0.167	-19.7*
Hebrew	0.248 /0.195	25.1*	0.760 /0.195	146.7*	0.096 /0.194	-32.5*	0.192 /0.195	-1.0	0.051 /0.195	-95.6*	0.193 /0.195	-1.0
Arabic	0.188 /0.160	8.3*	0.673 /0.160	86.6*	0.091 /0.160	-10.5*	0.151 /0.160	-1.8	0.033 /0.160	-60.4*	0.165 /0.160	1.3
Hungarian	0.182 /0.194	-2.9*	0.728 /0.193	70.9*	0.143 /0.195	-6.1*	0.220 /0.195	4.5*	0.056 /0.195	-38.9*	0.261 /0.196	11.0*
Persian	0.271 /0.216	19.0*	0.682 /0.216	111.0*	0.126 /0.216	-20.0*	0.220 /0.216	1.6	0.071 /0.216	-64.8*	0.242 /0.216	7.9*
Czech	0.185 /0.231	-10.1*	0.737 /0.231	68.7*	0.116 /0.231	-11.4*	0.237 /0.231	1.0	0.054 /0.231	-34.9*	0.284 /0.231	8.0*
Romanian	0.176 /0.143	7.2*	0.657 /0.142	60.5*	0.086 /0.142	-5.6*	0.212 /0.144	8.8*	0.020 /0.143	-40.4*	0.147 /0.143	0.6

* 2-sided p-value < 0.01

The construct the baseline, we first matched every revert in the network *ij* with another revert randomly selected from the set of reverts that *i* participated in (either as a reverter or reverted) within a time window of 24 hours. We then repeatedly sampled from this set of matched pairs. The set of samples was used to estimate the *Z*-score for the observed proportion of same-article interactions.

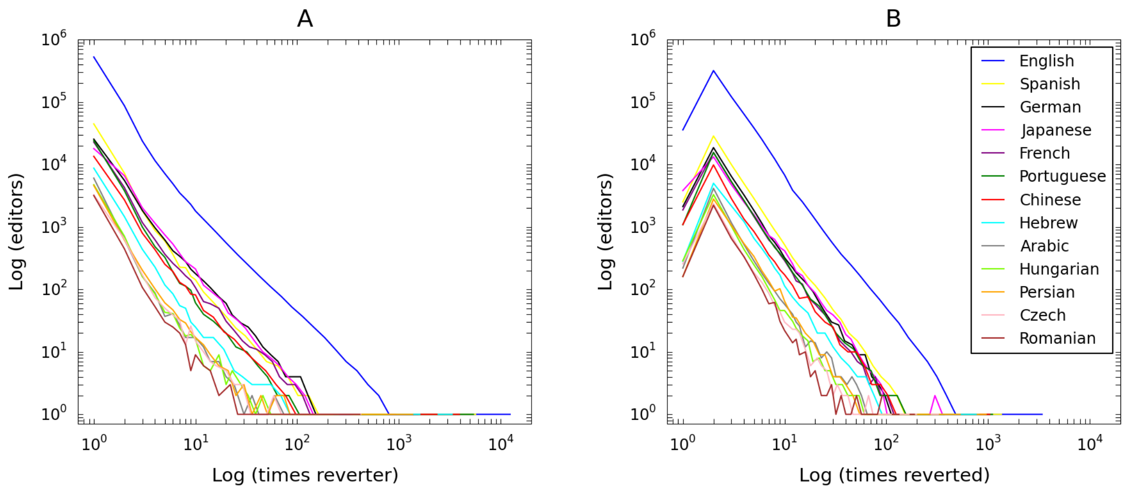

Fig. S1. Out-degree (A) and in-degree (B) distributions in the 13 networks of reverts.

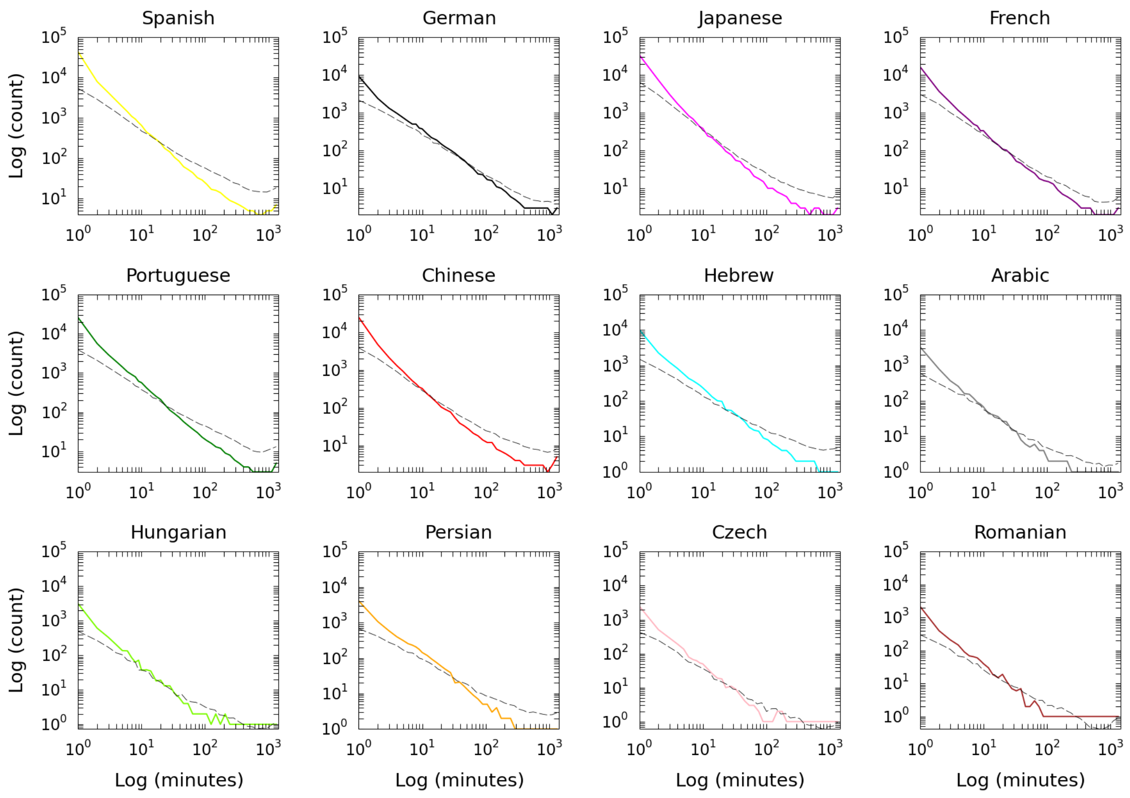

Fig. S2. Expected and observed counts of the *AB*-*AB* motif for 12 different language editions of Wikipedia. The dashed lines show the expected distributions according to the null model. Error bars are not shown as they are too small.

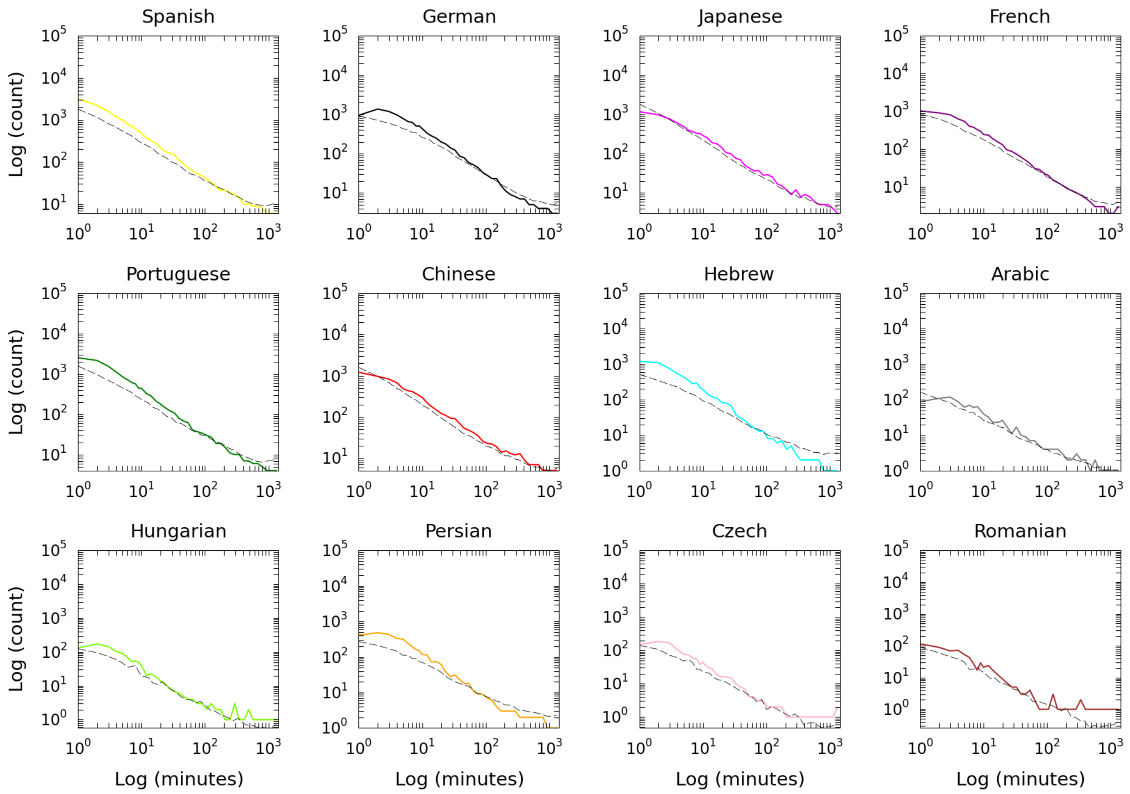

Fig. S3. Expected and observed counts of the *AB-BA* motif for 12 different language editions of Wikipedia. The dashed lines show the expected distributions according to the null model. Error bars are not shown as they are too small.

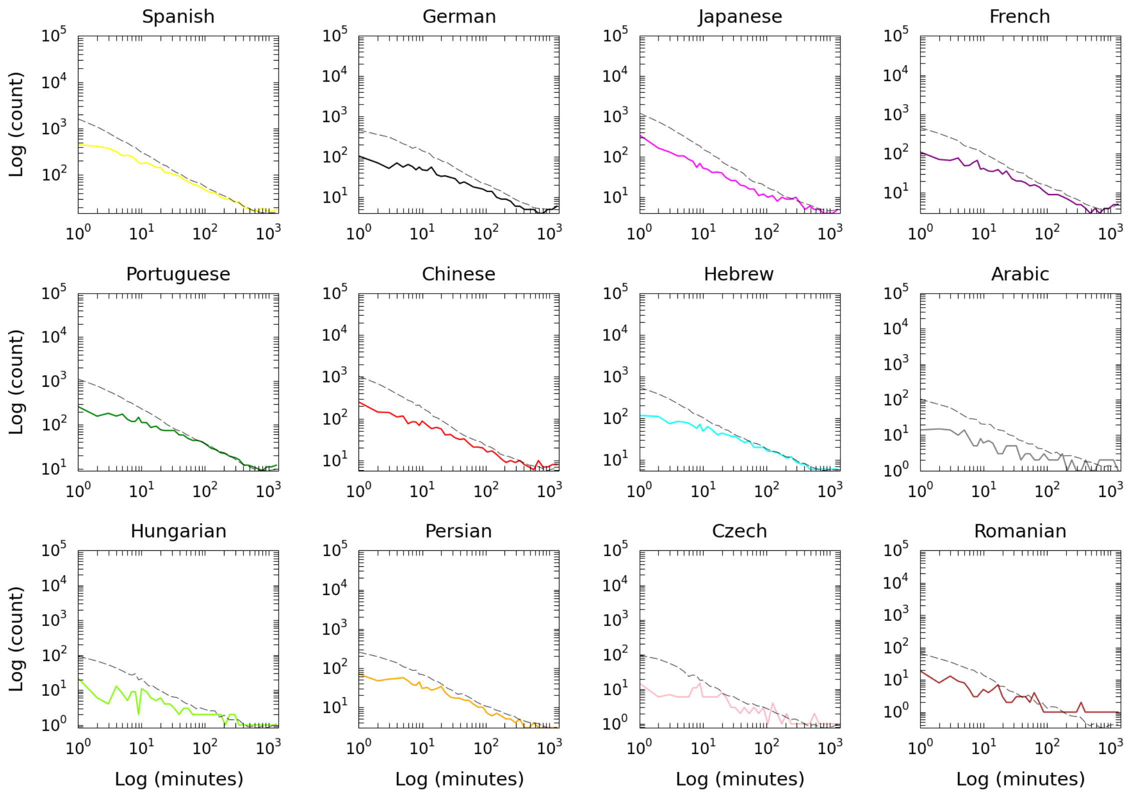

Fig. S4. Expected and observed counts of the *AB-BC* motif for 12 different language editions of Wikipedia. The dashed lines show the expected distributions according to the null model. Error bars are not shown as they are too small.

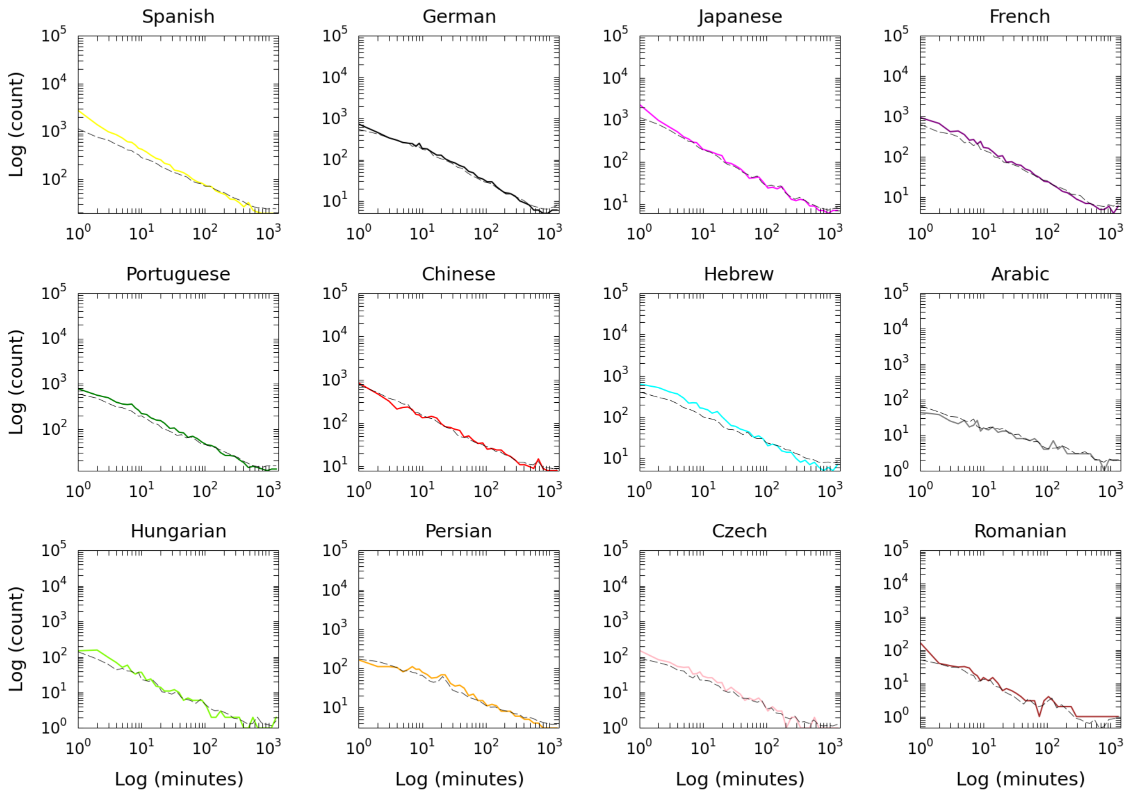

Fig. S5. Expected and observed counts of the *AB-CB* motif for 12 different language editions of Wikipedia. The dashed lines show the expected distributions according to the null model. Error bars are not shown as they are too small.

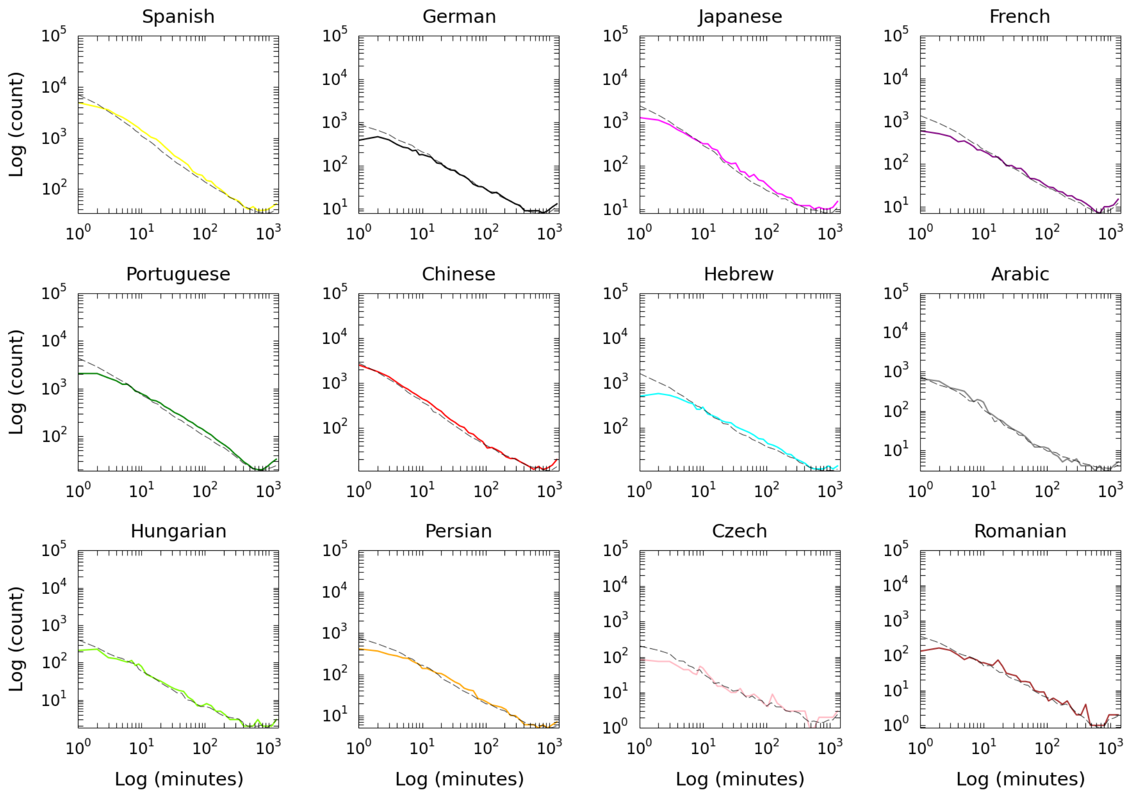

Fig. S6. Expected and observed counts of the *AB-AC* motif for 12 different language editions of Wikipedia. The dashed lines show the expected distributions according to the null model. Error bars are not shown as they are too small.

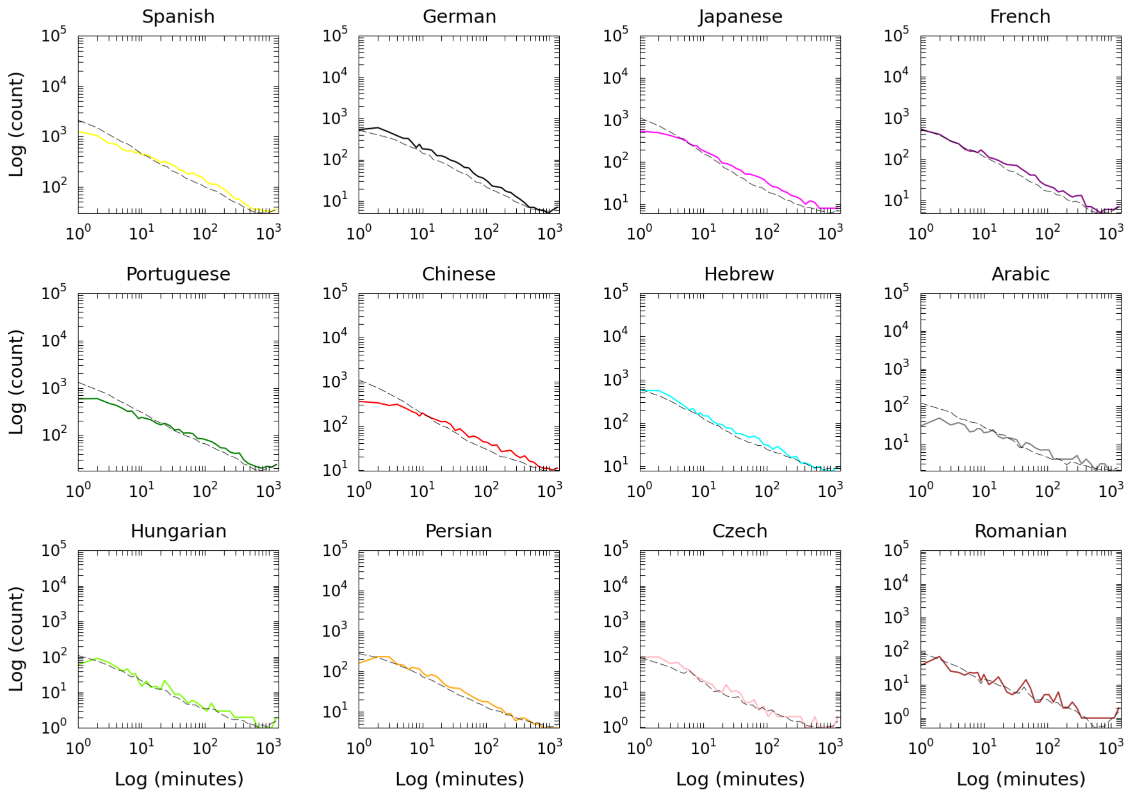

Fig. S7. Expected and observed counts of the *AB-CA* motif for 12 different language editions of Wikipedia. The dashed lines show the expected distributions according to the null model. Error bars are not shown as they are too small.

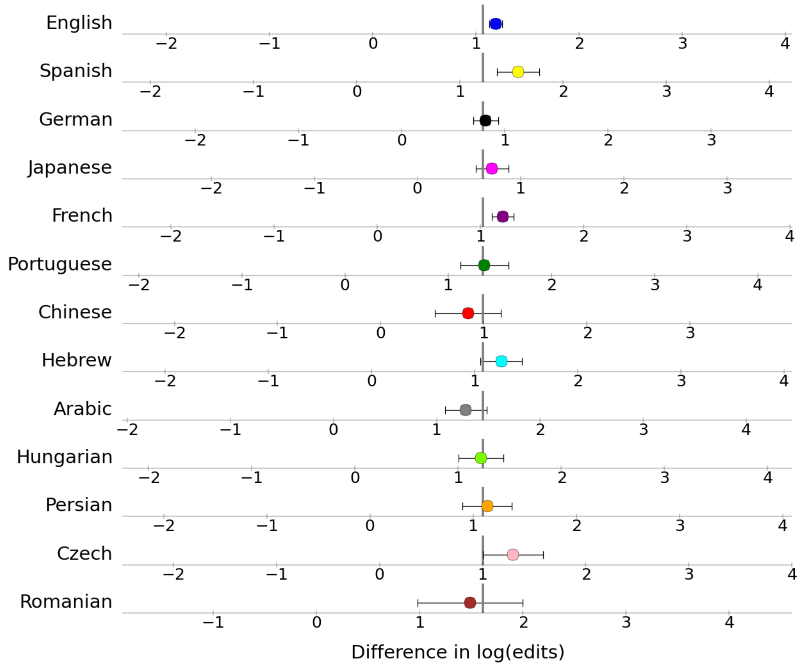

Fig. S8. Expected and observed difference in status between *A* and *B* for the *AB-AB* motif. Gray vertical lines show the expectation. Confidence intervals show 2.6 standard errors, corresponding to $p < 0.01$.

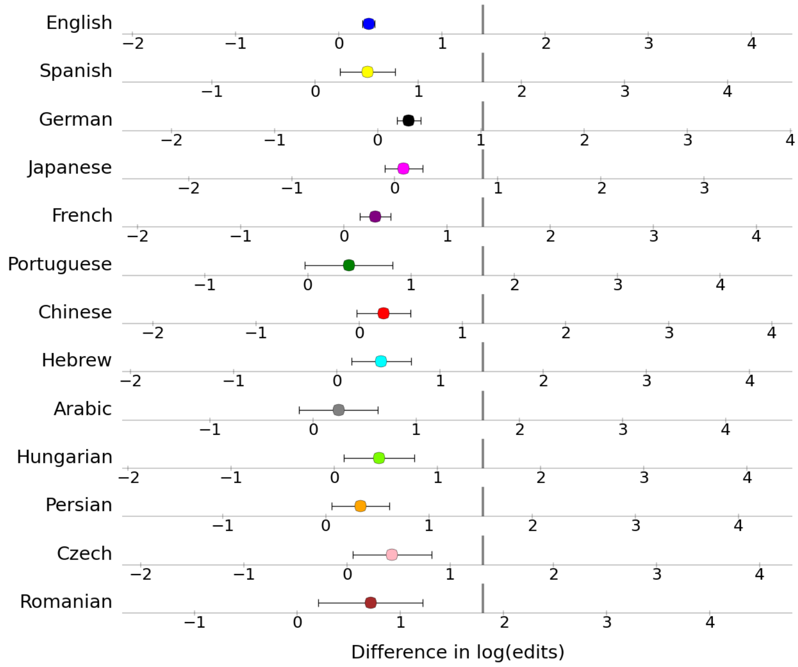

Fig. S9. Expected and observed difference in status between *A* and *B* for the *AB-BA* motif. Gray vertical lines show the expectation. Confidence intervals show 2.6 standard errors, corresponding to $p < 0.01$.

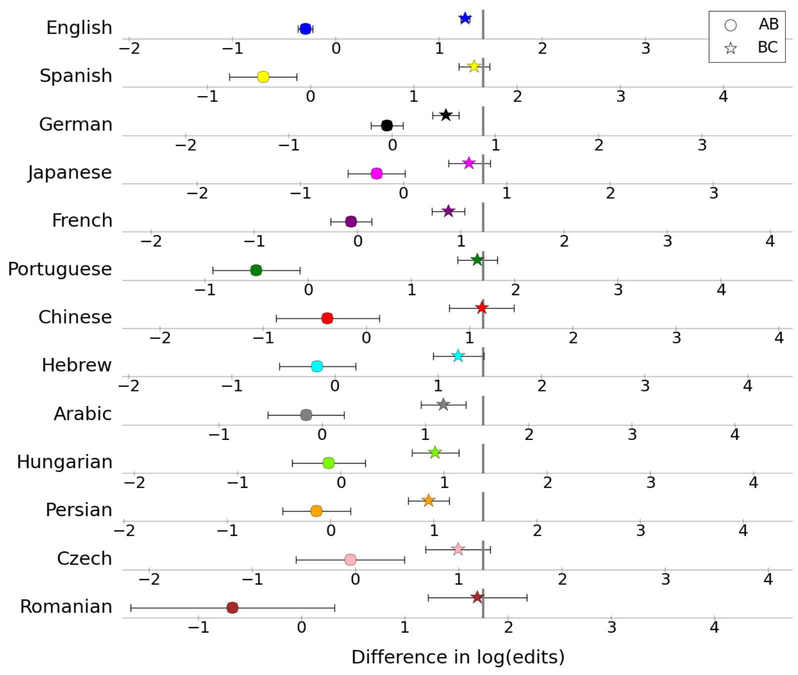

Fig. S10. Expected and observed difference in status between *A* and *B* (circles) and *B* and *C* (stars) for the *AB-BC* motif. Gray vertical lines show the expectation. Confidence intervals show 2.6 standard errors, corresponding to $p < 0.01$.

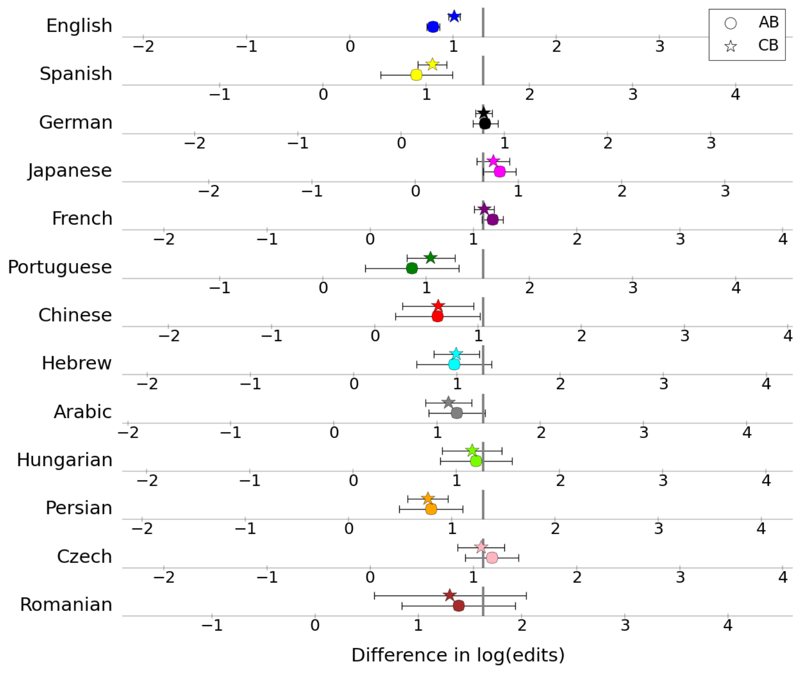

Fig. S11. Expected and observed difference in status between *A* and *B* (circles) and *C* and *B* (stars) for the *AB-CB* motif. Gray vertical lines show the expectation. Confidence intervals show 2.6 standard errors, corresponding to $p < 0.01$.

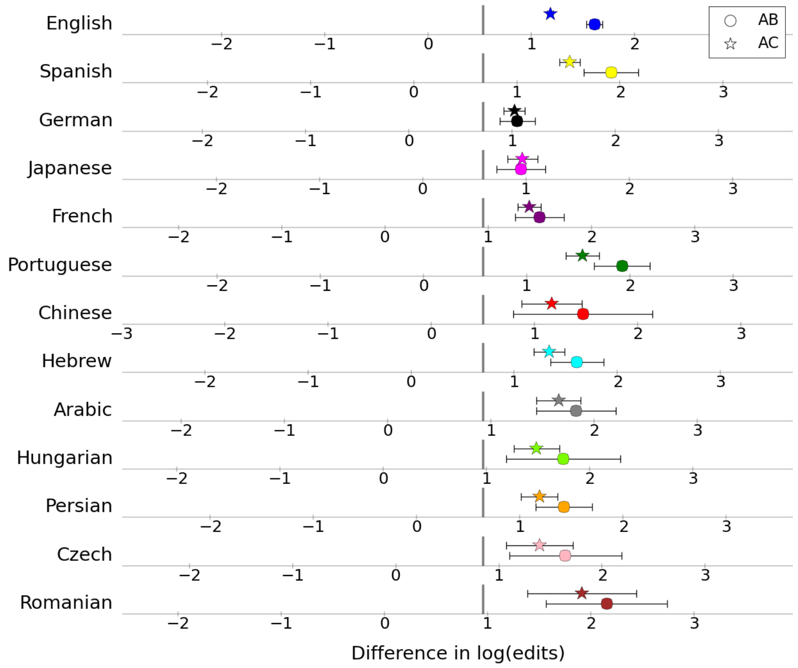

Fig. S12. Expected and observed difference in status between *A* and *B* (circles) and *A* and *C* (stars) for the *AB*-*AC* motif. Gray vertical lines show the expectation. Confidence intervals show 2.6 standard errors, corresponding to $p < 0.01$.

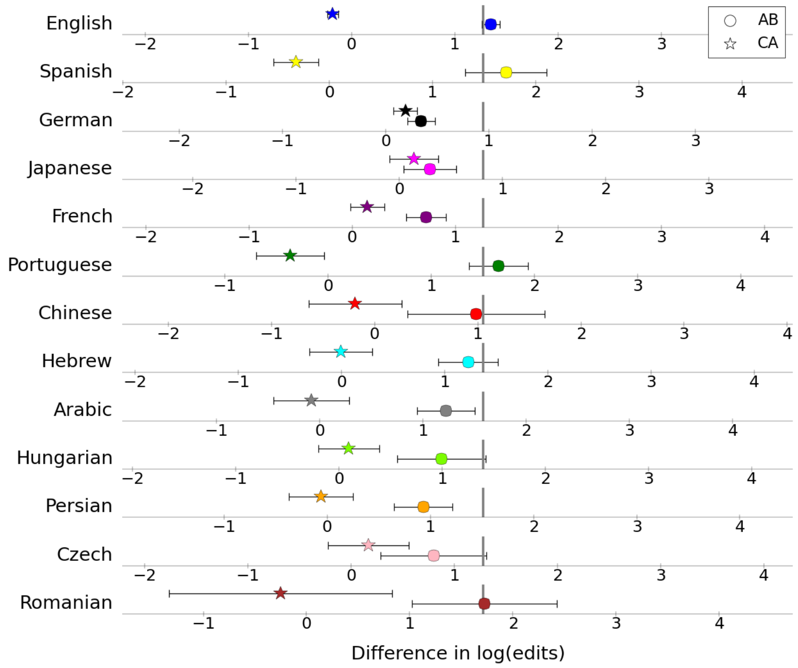

Fig. S13. Expected and observed difference in status between *A* and *B* (circles) and *C* and *A* (stars) for the *AB*-*CA* motif. Gray vertical lines show the expectation. Confidence intervals show 2.6 standard errors, corresponding to $p < 0.01$.

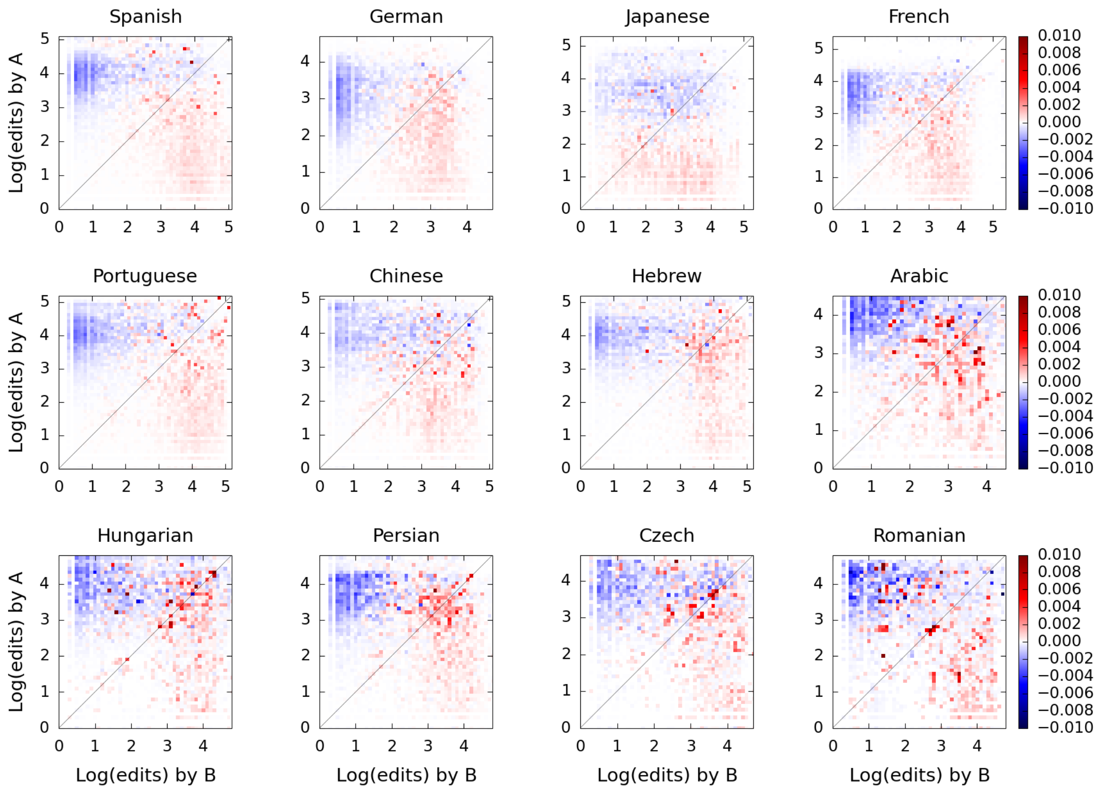

Fig. S14. Difference between the observed and expected distribution of status of *A* and *B* for the *AB-BA* motif.

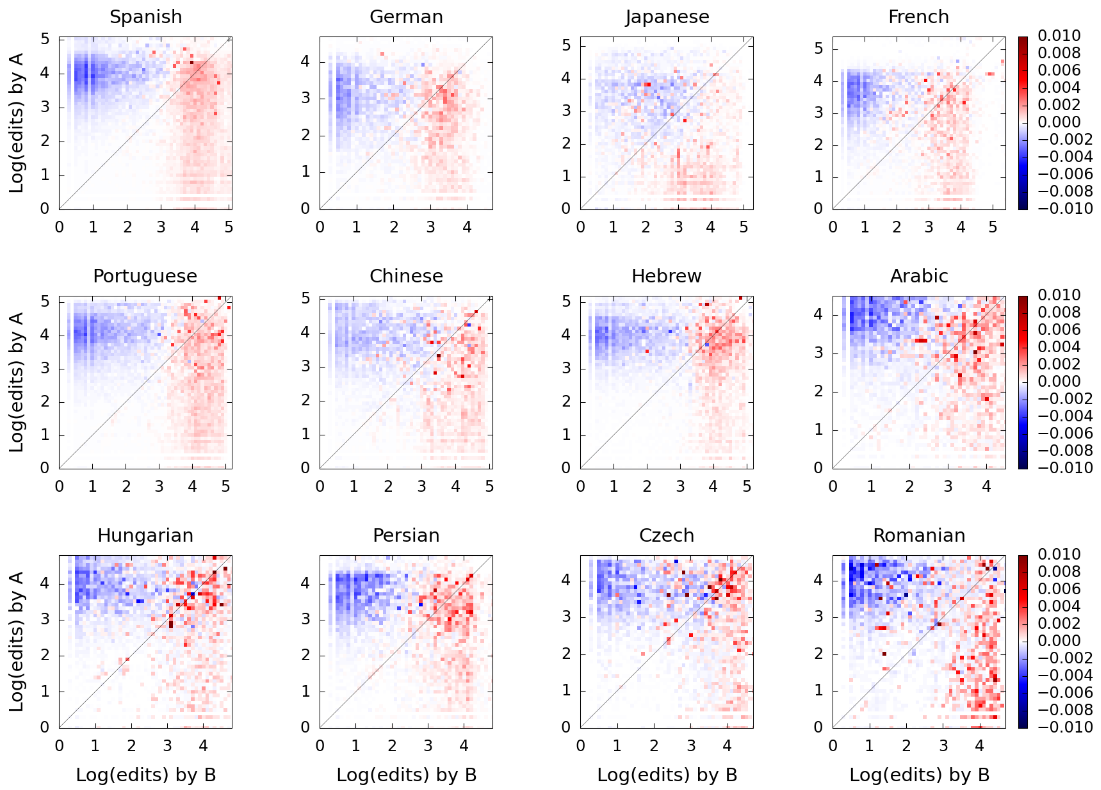

Fig. S15. Difference between the observed and expected distribution of status of *A* and *B* for the *AB*-*BC* motif.

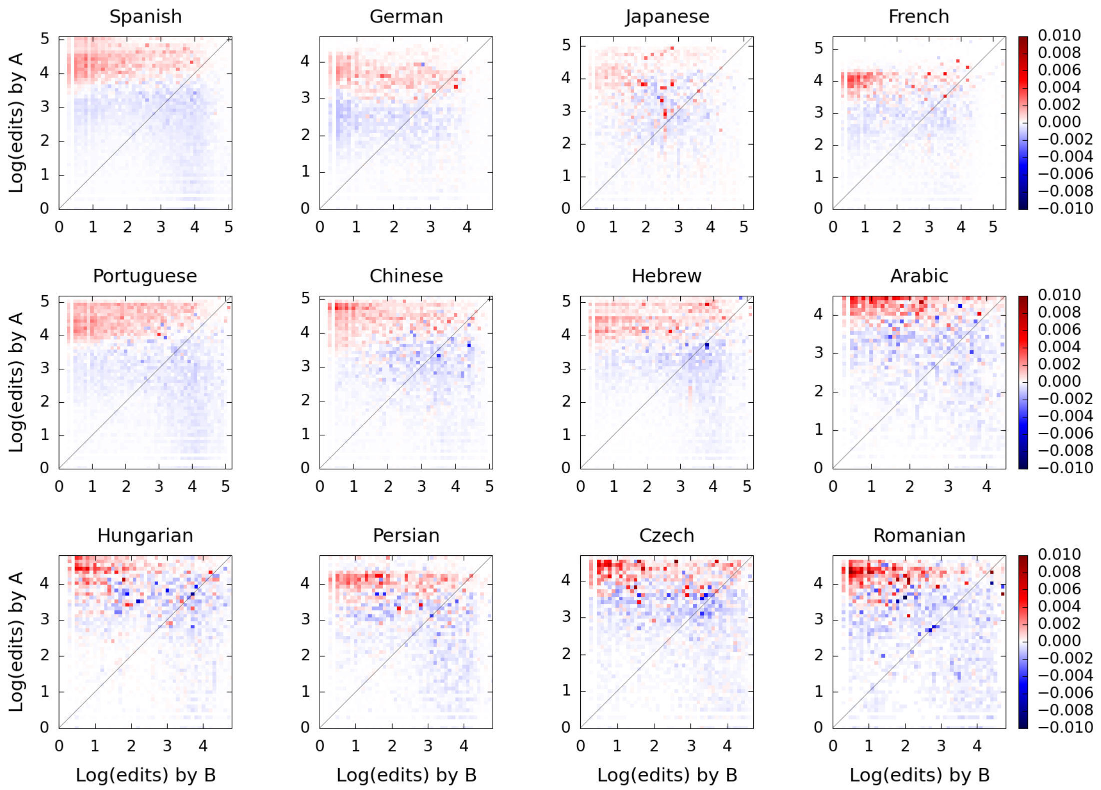

Fig. S16. Difference between the observed and expected distribution of status of *A* and *B* for the *AB*-*AC* motif.

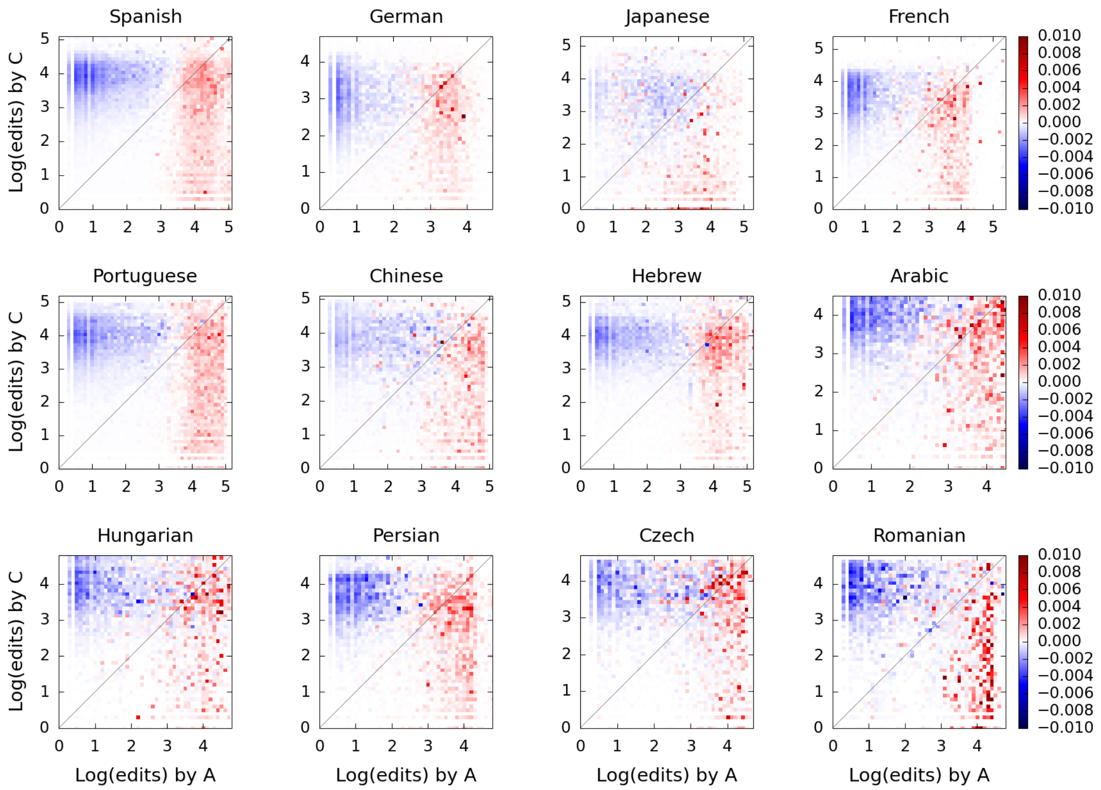

Fig. S17. Difference between the observed and expected distribution of status of *C* and *A* for the motifs associated with the *AB*-*CA* motif.

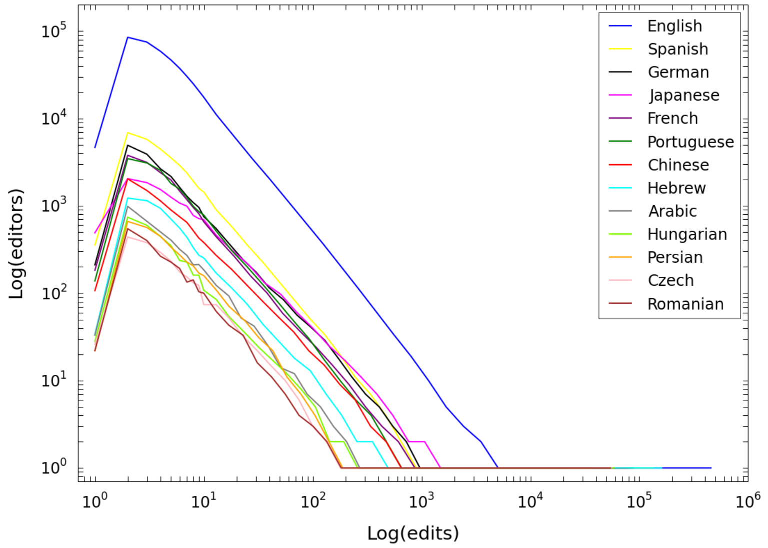

Fig. S18. Distribution of number of edits.
